# Multivitamin and Mineral Supplementation Containing Phytonutrients Scavenges Reactive Oxygen Species in Healthy Subjects: A Randomized, Double-Blinded, Placebo-Controlled Trial

**DOI:** 10.3390/nu11010101

**Published:** 2019-01-05

**Authors:** Seunghee Kang, Yeni Lim, You Jin Kim, Eun Sung Jung, Dong Ho Suh, Choong Hwan Lee, Eunmi Park, Jina Hong, Rodney A. Velliquette, Oran Kwon, Ji Yeon Kim

**Affiliations:** 1 Department of Nutritional Science and Food Management, Ewha Womans Universiy, Seoul 03760, Korea; nutrishee@gmail.com (S.K.); ynlim@ewha.ac.kr (Y.L.); eugenekim@ewha.ac.kr (Y.J.K.); 2 Department of Systems Biotechnology, Konkuk University, Seoul 05029, Korea; jes708@konkuk.ac.kr; 3 Department of Bioscience and Biotechnology, Konkuk University, Seoul 05029, Korea; sdh14031988@naver.com (D.H.S.); chlee123@konkuk.ac.kr (C.H.L.); 4 Department of Food and Nutrition, Hannam University, Daejeon 34430, Korea; eunmi_park@hnu.kr; 5 Access Business Group International, LLC, 5600 Beach Blvd., Buena Park, CA 90621, USA; jina.hong@amway.com (J.H.); rod.velliquette@amway.com (R.A.V.); 6 Department of Food Science and Technology, Seoul National University of Science and Technology, Seoul 01811, Korea

**Keywords:** phytonutrients, ROS scavenging, DNA damage, antioxidant capacity, human clinical study

## Abstract

Phytonutrients and vitamin and mineral supplementation have been reported to provide increased antioxidant capacity in humans; however, there is still controversy. In the current clinical trial, we examined the antioxidant and DNA protection capacity of a plant-based, multi-vitamin/mineral, and phytonutrient (PMP) supplementation in healthy adults who were habitually low in the consumption of fruits and vegetables. This study was an eight-week, double-blind, randomized, parallel-arm, and placebo-controlled trial. PMP supplementation for eight weeks reduced reactive oxygen species (ROS) and prevented DNA damage without altering endogenous antioxidant system. Plasma vitamins and phytonutrients were significantly correlated with ROS scavenging and DNA damage. In addition, gene expression analysis in PBMC showed subtle changes in superoxide metabolic processes. In this study, we showed that supplementation with a PMP significantly improved ROS scavenging activity and prevented DNA damage. However, additional research is still needed to further identify mechanisms of actions and the role of circulating phytonutrient metabolites.

## 1. Introduction

Clinical and epidemiological studies have shown that oxidative stress is related to cardiovascular disease, cancer, and other chronic diseases that account for the majority of mortality [1,2,3]. Several studies have shown that multivitamin and mineral supplements can help provide essential nutrients, maintain health, reduce the risk of various diseases, and support normal functions [4,5,6,7,8,9]. However, there are several clinical trial publications reporting no health benefits of multivitamin and mineral supplementation [10,11,12,13].

Besides vitamins and minerals, plants contain a wide variety of phytonutrients that have been reported to be involved in the prevention of chronic diseases [14]. Unlike vitamins and minerals, some phytonutrients (i.e., polyphenols) are known to be poorly absorbed in the small intestine [15]. Polyphenols are typically found in low concentrations (nmol/L to mmol/L range) in both plasma and urine [16], and detection requires sensitive analytical tools to have reliable and reproducible quantification. We previously showed that the contribution of phytonutrients to total antioxidant capacities was relatively higher than that of vitamins alone [17]. In another study, we reported that dietary supplementation with a phytonutrient-containing, multivitamin/mineral increased plasma folate level and folate metabolism in subjects with habitually low intake of fruits and vegetables [18]. In addition, the subjects in this study showed resistance to DNA damage while maintaining endogenous oxidative defense capacity [19]. However, the direct relation between plant phytonutrients and ROS scavenging and mechanisms of actions have not been completely elucidated.

In the current clinical trial, we examined the antioxidant and DNA protection capacity of a plant-based, multivitamin/mineral, and phytonutrient supplementation in healthy adults who were habitually low in the consumption of fruits and vegetables. To recruit subjects who were in low consumption of fruit and vegetable, recommended food score (RFS) was used [20]. RFS was validated to have relation with antioxidant capacity and, in our previous study, subjects with low RFS improved DNA repair by antioxidant nutrients [19]. In addition, correlations between antioxidant capacities and plasma vitamins and phytonutrients, and potential antioxidant mechanisms based on gene array and network analysis are reported.

## 2. Materials and Methods

### 2.1. Study Product

A plant-based, multivitamin/mineral and phytonutrient supplement (PMP) and a color-matched placebo were provided by Access Business Group International, LLC (Buena Park, CA, USA). The PMP supplement (12 tablets) contained the following micronutrients: 14 vitamins (700 μg retinol equivalents A, 2.4 mg B1, 2.8 mg B2, 3 mg B6, 4.8 μg B12, 200 mg C, 10 μg D, 22 mg α-tocopherol equivalents E, 55 μg K, 3 mg β-carotene, 60 μg biotin, 500 μg folate, 30 mg niacin, and 10 mg pantothenic acid), and 10 minerals (700 mg calcium, 50 μg chromium, 0.4 mg copper, 75 μg iodine, 6 mg iron, 3 mg manganese, 220 mg magnesium, 25 μg molybdenum, 55 μg selenium and 12 mg zinc). The PMP supplement also contained phytonutrients from extracts or powders of acerola, alfalfa, black currant, blueberry, elderberry, grape, grapefruit, kelp, lemon, mandarin orange, marigold, onion, orange, parsley, peppermint, rosemary, spinach, tomato, turmeric, and watercress, and quercetin granular. Most phytonutrients were from botanical extracts, i.e., botanical feedstocksthat were further concentrated by removing other parts of the pant structure while preserving the phytonutrients. A few ingredients were from dehydrates, i.e., dried botanical feedstock powders in which all water was removed from the plant while all other parts of the plant (e.g., fiber, cell walls, sugars, and phytonutrients) remain. The placebo sample for this study was formulated to match the shape and color of the PMP tablets. The placebo was composed of microcrystalline cellulose, silicon dioxide, magnesium stearate and colorants.

### 2.2. Subjects

**Figure 1:**
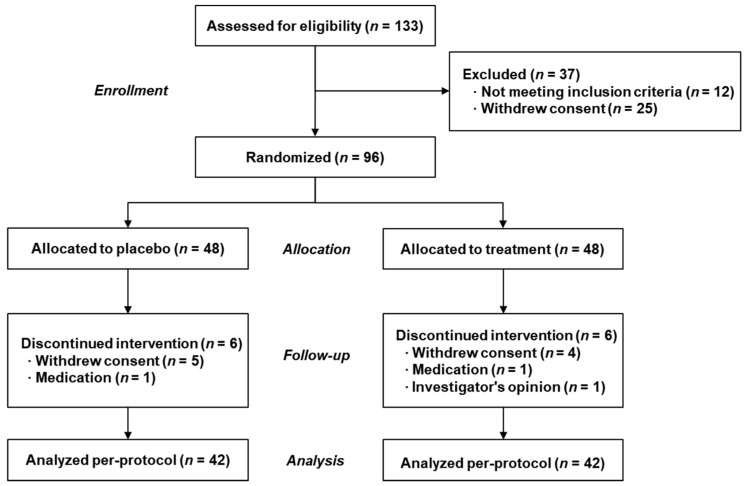
CONSORT flow diagram summarizing the subject’s disposition for the study.

Healthy adults (25–69 years old) with habitually low fruit and vegetable intake as determined by RFS ≤ 36 (scale, 0–47) [20] and body fat ≥ 20% (InBody; Biospace, Seoul, Korea) were eligible. All inclusion and exclusion criteria are presented in Table S1. One hundred thirty-three subjects were recruited from Ewha Womans University (Seoul, Korea). Twelve subjects were excluded for not meeting the eligible criteria, 25 subjects withdraw their consent, and the remaining 96 subjects were enrolled in the trial and received the baseline assessment. There are presented a Consolidated Standards of Reporting Trials (CONSORT) flow diagram in Figure 1 [21]. All subjects provided written informed consent before enrollment. The study protocol was approved by the Institutional Review Boards of Ewha Womans University (IRB No.119-16) and registered in the International Clinical Trials Registry Platform of the WHO (KCT0002055).

### 2.3. Study Design

The clinical trial was an eight-week, double-blind, randomized, parallel-arm, and placebo-controlled study. During the two-week run-in and eight-week trial, subjects were instructed by dietitians to maintain dietary and lifestyle habits, including alcohol intake, physical activity, and sleep time but to avoid certain foods that ranked rich in antioxidant nutrients. After the two-week run-in period, subjects were randomly assigned to either the placebo (*n* = 48) or PMP (*n* = 48) group and balanced by age and gender (Figure S1). The subjects were assigned to take six tablets, twice daily, for a total of 12 tablets per day during the eight-week period, preferably with water in the middle of a meal. Remaining tablets were counted to assess compliance at Week 4 and 8 during site visits. To assess nutrient intake, lifestyle, and monitor dietary compliance during the trial, subjects were given dietary instructions and provided a three-day diet record (two weekdays and one weekend) at baseline, and Week 4 and 8 using a smart phone application. On the evening prior to each blood draw (baseline and week 8), subjects consumed a standardized meal, to reduce the potential confounding effect of previous meal. The standardized meal (approximately 640 kcal; carbohydrate:protein:lipid % ratio = 65:23:12) consisted of steamed rice (340 kcal in 170 g) and Bulgogi (roasted beef marinated with soybean sauce; 300 kcal in 170 g). After a 12 h overnight fast, venous blood was collected in tubes with ethylenediaminetetraacetic acid (EDTA). The plasma, and erythrocytes were separated by centrifuge at 4 °C (1500× *g* for 10 min). Peripheral blood mononuclear cells (PBMC) were isolated from whole blood by density centrifugation using Histopaque^®^-1077 reagent (Sigma-Aldrich, St. Louis, MO, USA) according to manufacturer’s instructions. All samples were stored at −80 °C until analyzed. The site visit schedule is illustrated in Figure S1.

### 2.4. Oxidative Stress-Related Biochemical Analysis

Plasma reactive oxygen species (ROS) level was measured by luminol amplified chemiluminescence (5-amino-2,3-dihydro-1,4-phtha-zinedione, Sigma-Aldrich, St. Louis, MO, USA) using a chemiluminescence Fluoroskan Ascent FL (Thermo Fisher, Vantaa, Finland) at 37 °C [22]. In brief, 50 µL of plasma and 200 µL of 2 mM luminol (dissolved in 0.05 M NaOH solution) were pipetted into a 96-well microplate (SPL Life Sciences Co., Ltd., Pocheon-si, Gyeonggi-do, Korea), and then read for 1 min to measure background. Then, 100 µL of 10 mM H_2_O_2_ was added and light emission was analyzed for 15 min at 1 min intervals. After adjusting for background levels, area under the curve (AUC) of ROS was calculated by the trapezoidal rule.

Comet assay was performed using a single-cell gel electrophoresis assay as previously described [23]. In brief, PBMC were resuspended in 2 mL of cold phosphate-buffered saline to a concentration of 1 × 10^5^ cells/mL using an automated cell counter (TC 10^TM^; BIO-RAD Laboratories, Inc., Hercules, CA, USA). Fifty microliters was mixed with 150 µL of 1% low-gelling-temperature agarose (Sigma-Aldrich, St. Louis, MO, USA), and immediately spread on Comet slides (Trevigen Inc., Gaithersburg, MD, USA) and incubated at 4 °C for 30 min. Slides were then submerged in a lysis solution (2.5 M NaCl, 100 mM Na2EDTA, 10 M Trizma-base, 1% (*v*/*v*) Triton X-100, and 10% (*v*/*v*) DMSO, pH 10) for 1 h at 4 °C. The slides were transferred to prechilled alkaline electrophoresis solution (10 N NaOH, 200 mM EDTA, pH > 13) and then electrophoresed at 31 V for 30 min at 4 °C. At the end of the electrophoresis, the slides were immersed in distilled water twice for 10 min, then 5 min in 70% ethanol, and dried overnight at room temperature. DNA was stained with 50 μL of SYBR Green I dye (Sigma-Aldrich, St. Louis, MO, USA) that was diluted 1:10,000 in Tris–EDTA buffer (pH 7.5), for 5 min at 4 °C, and visualized with a fluorescence microscope (Nikon, Tokyo, Japan) at 4× magnification. Images from 50 comets on each slide were analyzed with COMET VI image analysis software (Perceptive Instruments, Suffolk, UK).

Total malondialdehyde (MDA) levels in plasma were quantified by high-performance liquid chromatography-fluorescence detection system (HPLC-FLD; Shiseido Co, Ltd., Tokyo, Japan). Fifty microliters of plasma were mixed with 300 μL of 0.44 M phosphoric acid (Duksan Pure Chemicals Co., Ltd., Ansan-si, Gyeonggi-do, Korea) and 150 μL of 42 mM 2-thiobarbituric acid (TBA; Sigma-Aldrich, St. Louis, MO, USA). After incubating plasma samples at 95 °C for 1 h, samples were cooled to 4 °C followed by centrifugation at 2500× *g* for 3 min. The supernatants were filtered with a 0.45 µm PTFE Syringe filter (Woongki Science, Seoul, Korea). Ten microliters of the TBA-MDA adduct were injected into a Capcell Pak C18 column (UG120 type, 4.6 mm i.d. × 250 mm, 5 μm particle size; Shiseido Co, Ltd., Tokyo, Japan). The mobile phase was 50 mM phosphate:methanol buffer (7:3 *v*/*v*, pH 6.8), at an isocratic flow rate of 1 mL/min. The MDA levels were determined from a standard curve using 1,1,3,3-Tetraethoxypropane (Sigma-Aldrich, St. Louis, MO, USA).

Plasma oxidized low-density lipoprotein (Ox-LDL) was measured using a sandwich enzyme-linked immunosorbent assay (ELISA) kit according to the manufacturer’s instruction (Mercodia, Uppsala, Sweden). The level of reduced glutathione (GSH), oxidized glutathione (GSSG), and antioxidant enzyme activities (superoxide dismutase (SOD) and glutathione peroxidase (GPx)) in erythrocytes were measured spectrophotometrically using commercially available kits (Cayman, Ann Arbor, MI, USA) following the manufacturer’s instructions.

### 2.5. Western Blot

Plasma lysates were prepared and Western blot was performed as described previously [24]. Briefly, plasma from ten subjects from each group was prepared by lysing in radioimmunoprecipitation assay buffer (50 mmol/L Tris, pH7.3, 150 mmol/L NaCl, 1 mmol/L EDTA, 1% Triton X-100, 0.5% Na-deoxycholate, and 0.1% SDS) with protease inhibitors, NaVO_4_ and NaF. One hundred micrograms of plasma lysates were resolved in 10% SDS-PAGE and then transferred to PVDF membranes. Equal loading and transfer of proteins was verified by Ponceau red staining of the membranes. Blots were incubated using the following antibodies: anti-pCHK1 (Ser345, 1:500 dilution, Cell Signaling Technology, Danvers, MA, USA) and anti-β-actin (1:5000 dilution, Cell Signaling Technology, Danvers, MA, USA). Proteins were detected by chemiluminescence detection (Pierce ECL Western blot substrate, Thermo Fisher Scientific Inc., Rockford, IL, USA) and analyzed by FUSION Solo (Vilber Lourmat, Collégien, France).

### 2.6. Vitamin and Phytonutrient Analysis

Before analysis of plasma phytonutrients, a chemical fingerprint of the PMP study product was generated with ultra-performance liquid chromatography-quadrupole/time-of-flight mass spectrometry (UPLC-Q-TOF-MS) [25] and ultrahigh-performance liquid chromatography-linear trap quadrupole-ion trap tandem-mass spectrometry (UHPLC-LTQ-IT-MS/MS) [26]. In brief, six tablets were pulverized in a mortar and pestle, extracted with 1 mL of methanol and mixed for 1 h, then centrifuged at 2370× *g* for 5 min at 4 °C. The supernatants were then filtered through a 0.2 µm PTFE filter and evaporated with a speed vac (Modulspin 31; Biotron, Bucheon-si, Gyeonggi-do, Korea). Ten microliters (50 mg/mL *wt*/*v*) of each sample were injected into the LC-MS.

For analysis of plasma vitamins and phytonutrients, 200 μL of plasma taken from each subject were pooled into four samples that contained plasma from 12 subjects (due to limited plasma volume). Eight hundred microliters of plasma were extracted with 3.2 mL of methanol with a MM400 mixer mill (Retsch^®^, Haan, Germany) at a frequency of 30 s^−1^ for 10 min. After centrifugation at 4 °C (12,578× *g* for 10 min), supernatants were filtered through 0.2 µm PTFE filters, and then evaporated with a speed vac. The final concentration of each sample was 50 mg/mL (*wt*/*vol*).

LC-triple-Q-MS analysis was performed on Nexera2 LC system (Shimadzu Corp., Kyoto, Japan) combined with a triple quadrupole MS equipped with an electrospray source (LC-MS 8040, Shimadzu). Five microliters were injected into a Kinetex C18 column (100 × 2.1 mm, 2.6 μm, Phenomenex, Torrance, CA, USA) with a mobile phase containing 0.1% formic acid (solvent A) and acetonitrile containing 0.1% formic acid (solvent B) at a flow rate of 300 μL/min. The gradient was 5% solvent B for 1 min, and linearly increased from 5% to 100% over 9 min, and then decreased to 5% for 1 min. The MS was operated under the following conditions: capillary voltage −3000 V, capillary temperature 350 °C, vaporizer temperature 300 °C, sheath gas 3 L/min, ion sweep gas 2.0 Arb, Aux gas 10 Arb, and drying gas 8 L/min. The following multiple reaction monitoring transitions used were: 220 > 95 for pantothenic acid, 175 > 115 for ascorbic acid, 442 > 295 for folic acid, 359 > 161 for rosmarinic acid, 609 > 301 for hesperidin, 387 > 206 for tuberonic acid glucoside, 595 > 287 for cyanidin 3-*O*-glucoside, 609 > 301 for rutin, 593 > 285 for kaempferol-rutinoside, 463 > 301 for quercetin 3-*O*-glucoside, 625 > 463 for quercetin-diglucoside, 579 > 271 for naringin, 301 > 151 for quercetin, 463 > 301 for peonidin 3-glucoside, 337 > 119 for demethoxycurcumin, 367 > 217 for curcumin, 283 > 268 for wogonin, 418 > 356 for gamma-tocopherol, 331 > 287 for carnosic acid, and 273 > 149 for phloretin.

### 2.7. Quantitative PCR Array on Peripheral Blood Mononuclear Cells (PBMC)

Quantitative polymerase chain reaction (qPCR) array was performed using AccuPower^®^ Customized qPCR Panel Kit (Bioneer, Daejeon, Korea) as described previously [27]. Briefly, total RNA was extracted using a TRIZOL reagent (Invitrogen Co., Carlsbad, CA, USA). The total RNA concentrations and the 260/280 nm ratio were evaluated using a spectrophotometer (Biospec-nano; Shimadzu Corp, Kyoto, Kyoto Prefecture, Japan). Only samples with a 260/280 nm ratio between 1.7 and 2.1 were processed further. cDNA was generated using an *AccuPower*^®^*RocketScript*^TM^Cycle RT PreMix (Bioneer, Daejeon, Korea). qPCR was performed with a Step-One-Plus RT-PCR system (Applied Biosystems, Foster City, CA, USA) in a 96-well microplate using a final volume of 20 μL of the following components: 1 μL of ROX dye, 1 μL of template, 10 μL of 2× GreenStar qPCR master mix, and 8 μL of nanofiltered water (Bioneer, Daejeon, Korea) per well. Amplifications were performed using a 10 min template predenaturation step at 95 °C, followed by 40 cycles of 95 °C for 5 s, 58 °C for 25 s, and 72 °C for 30 s. A total of 88 genes were classified into the following categories and are shown in Table S2: inflammatory mediators and signaling molecules (47 genes), plaque formation and coagulation (3 genes), antioxidant (14 genes), blood cell differentiation (2 genes), and lipid/lipoprotein metabolism (22 genes). The relative amounts of mRNA were normalized to glyceraldehyde 3-phosphate dehydrogenase (GAPDH), and the relative amount of RNA was calculated using the comparative C_T_ method.

### 2.8. Core Interaction Network of qPCR Analysis

The network-based enrichment analysis of selected genes that were upregulated or downregulated after PMP supplementation was performed using the EnrichNet database (http://enrichnet.org) at default settings [28]. Gene-gene interaction sub-network for selected gene set predicted to be functionally associated by gene ontology term. Each node represents a physical entity. Each edge represents a gene regulatory interaction.

### 2.9. Statistical Analysis

The sample size was estimated to be 48 subjects per group to provide an 80% power of demonstrating a significant difference in tail intensity, based on our previous study [19] and a drop-out rate of 20%. All data were analyzed on a per-protocol principle according to the pre-defined criteria for inclusion. All variables at each time point were tested for normal distribution with the Shapiro—Wilks test, and skewed data were normalized by square root transformation. Differences in the baseline characteristics between the placebo and PMP groups were assessed using the Student’s *t*-test for continuous variables and chi-square test for categorical variables. Differences in the means of outcomes were analyzed using a linear mixed-effects (LME) model, considering a random effect (participant), a random error (within-participant), fixed effects (group, week, and the interaction between group and week), and covariates. Age, gender, body mass index, RFS, total energy intake, smoking, alcohol, and dietary β-cryptoxanthin and flavanone intakes were used as covariates. Additionally, the pooled *p*-value was derived from multivariate linear mixed-effects model in the combined data of comet assay to the effect of PMP on the overall DNA damage. Before the analysis, each parameter was standardized using a z-score transformation by subtracting the mean and then dividing by the standard deviation. The Pearson correlation analysis was used to analyze the correlations between plasma vitamins and phytonutrients and biomarkers. All statistical analyses were performed using SAS 9.4 (SAS Institute, Cary, NC, USA) and *p*-value < 0.05 was considered significant.

## 3. Results

### 3.1. Subject Characteristics throughout the Study

Of 96 eligible subjects, 84 (42 in the placebo group and 42 in the PMP group) completed the eight-week supplementation for inclusion in the analysis (Figure 1). Nine subjects withdrew their consent for personal reasons, two and one subjects were excluded due to medication and investigator’s opinion, respectively. There was no significant difference in baseline characteristics between the two groups (Table 1). Participants in this study had RFS of 19.1 ± 1.3 for placebo and 19.1 ± 1.5 for PMP group, which was categorized as low consumers of fruits and vegetables. Lifestyles including amount of alcohol consumption, physical activity level, and total sleep time were unchanged during the study (Table S3). The mean compliance for all subjects was 92.8%. No serious or severe adverse events were observed.

### 3.2. DNA Oxidative Damage

**Figure 2:**
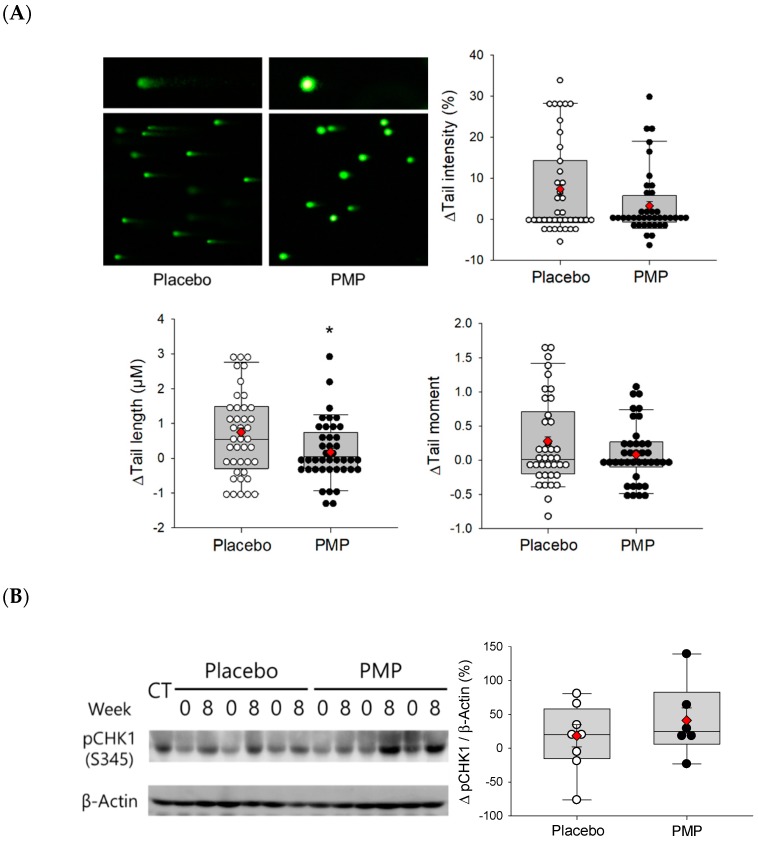
Effect of eight-week supplementation on DNA damage and repair: (**A**) Comet assay on PBMC; and (**B**) Representative Western blot of plasma phosphorylated checkpoint kinase 1 (pCHK1-Ser345). Change in DNA damage and repair mechanism are presented as a box and scatter plots. The ends of the box are the upper and lower quartiles. The medians are marked by a vertical line inside the boxes. The whiskers are the two lines outside the box that extend to the highest and lowest value. Dots are individual values of each subjects. For Western blot, representative samples (*n* = 8 for placebo and *n* = 6 for PMP) were analyzed. CT (a sample from the PMPgroup) indicates a loading control for analyzing quantitate data, 0 indicates before supplementation and 8 indicates after supplementation. Statistical significance of comet assay was determined by linear mixed-effects model. In the case of Western blot, linear mixed-effect model was used to compare the difference within each group (* *p* < 0.05). LS means (◆).

DNA damage in PBMC was significantly decreased for tail length after PMP supplementation compared to placebo group (*p* = 0.042) (Figure 2A). Although there were no significant differences in the changes of tail intensity and tail moment between placebo and PMP treated group, when all comet parameters were combined and analyzed using the multivariate linear mixed-effects model, pooled *p*-value was 0.032 in PMP compared to placebo. In addition, we evaluated DNA damage and repair response by measuring plasma phosphorylated checkpoint kinase 1 (pCHK1-Ser345) protein level after supplementation. Among 10 plasma samples for each group, eight samples from placebo and six samples for PMP group were used for Western blot analysis. After eight weeks of supplementation, pCHK1 protein level tended to increase in PMP group compared to baseline (Figure 2B; *p* = 0.091). Taken together, PMP supplementation induced the DNA repair cell signaling and increased DNA damage and repair response in plasma.

### 3.3. ROS Scavenging

**Figure 3:**
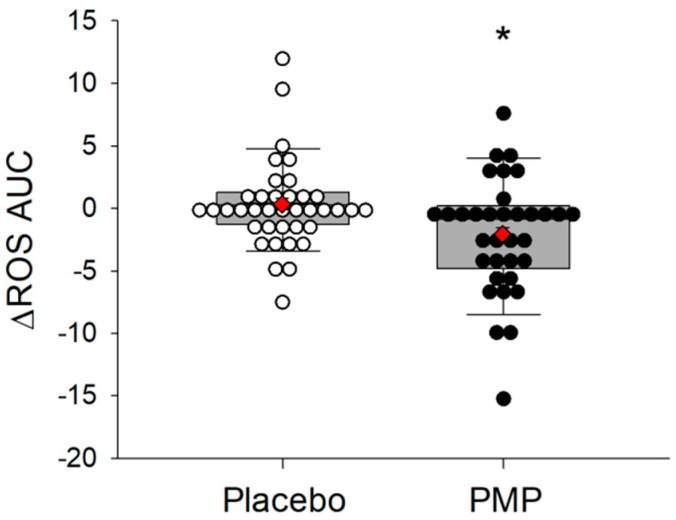
Box and scatter plot of the change in plasma ROS AUC after eight-week supplementation. ROS AUC was calculated by the trapezoidal rule. The ends of the boxes are the upper and lower quartiles. The median is marked by a vertical line inside the box. The whiskers are the two lines outside the box that extend to the highest and lowest value. Dots are individual values of each subjects. Statistical significance was determined by linear mixed-effects model (* *p* < 0.05). Reactive oxygen species (ROS); area under the curve (AUC); LS means (◆).

Luminol-dependent chemiluminescence was used to determine plasma ROS level following placebo and PMP supplement for eight weeks. PMP group had significantly lower ROS AUC compared to placebo group (*p* = 0.018) (Figure 3). However, other plasma biomarkers measured in this study such as MDA and Ox-LDL levels and erythrocyte SOD and GPx activity were not significantly different between the two groups (Table 2).

### 3.4. qPCR RNA Array Analysis

**Figure 4:**
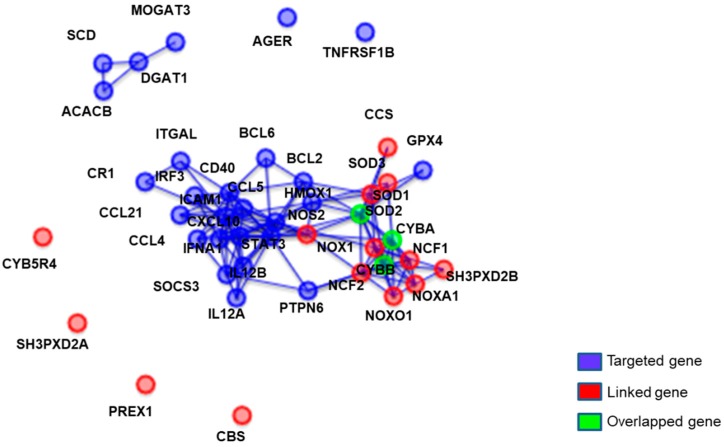
Network of up-regulated genes changed by eight-week PMP supplementation. Genes were selected based on differential expression pattern compared to placebo. Each node represents a physical entity. Each edge represents a gene regulatory interaction. Blue circles are genes that are targeted and analyzed by PCR analysis. Red circles are genes related process given by EnrichNet database (http://enrichnet.org), but not analyzed. Green circles denote overlapped gene between blue and red genes.

Of the 88 genes analyzed in the PCR array, 87 genes amplified, all except NOS2, however, there were no significant differences between the two groups in any of the genes (Table S4). Although no statistical significance was found, network-enrichment analysis was performed to analyze interactions between genes and explore biological pathways potentially modulated by the PMP supplementation. Genes that were not changed in placebo but changed in PMP supplementation were selected for network analysis. Among 87 genes, 52 changed over 20% in the placebo group, which were then removed from network analysis. Of the remaining 35 genes that showed no changes in placebo group, the PMP supplemented group showed up-regulation of 32 genes and down-regulation of 3 genes. Besides the genes measured in this study (blue and green circles), associated genes are shown (red circle) in Figure 4. Network-enrichment analysis with GO annotation system indicated that, among up-regulated genes, SOD2, CYBA, and CYBB are involved in superoxide metabolic processes (Figure 4). In addition, CCS, CBS, CYB5R4, NCF1, NCF2, NOS2, NOX, NOXA1, NOXO1, PREX1, SH3PXD2A, SH3PXD2B, SOD1, and SOD3 were associated with up-regulated genes, which are also genes involved with superoxide metabolic processes. In the GO pathway analysis, several pathways related to ROS such as superoxide metabolic pathway and superoxide anion generation were ranked in the Top 10 changed pathways with statistical significance (q < 0.05; Table S5).

### 3.5. Vitamin and Phytonutrient Measurement

**Figure 5:**
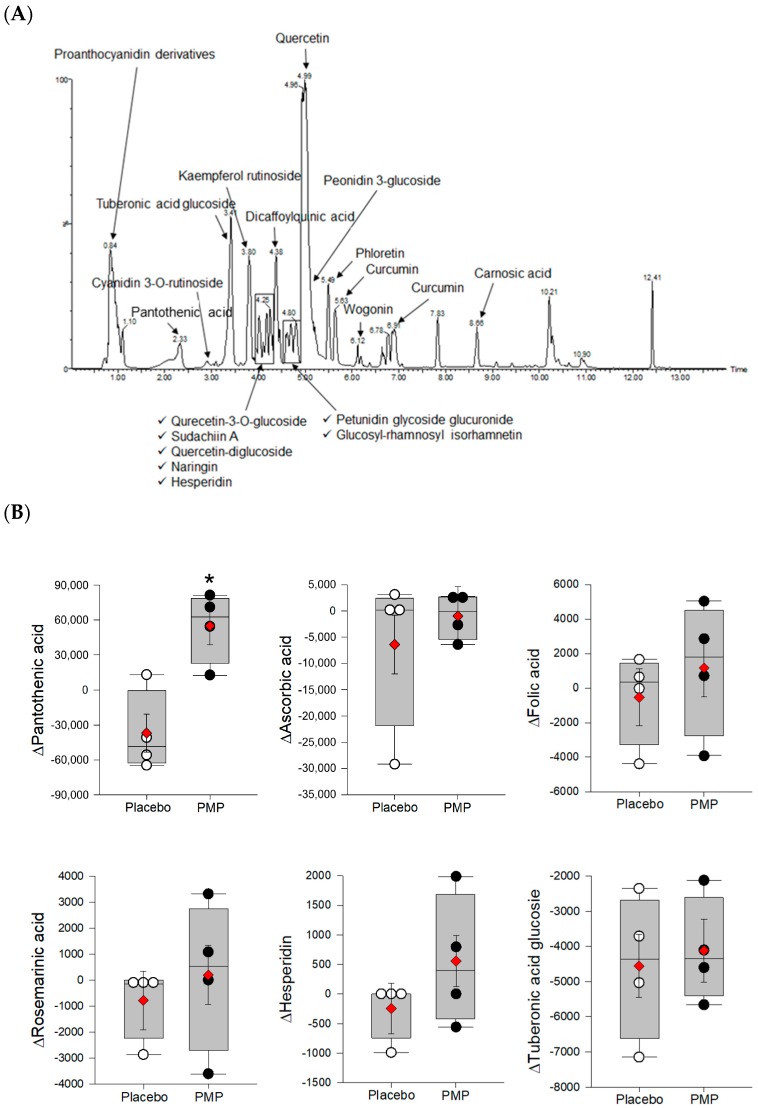
(**A**) The UPLC-Q-TOF-MS chromatographic fingerprint of the PMP study product; and (**B**) change in plasma after eight-week supplementation. The y-axis of box plots indicates the change in the relative peak area of each vitamin and phytonutrient. Statistical significances were determined by linear mixed-effects model (* *p* < 0.05). LSmeans (◆).

**Figure 6:**
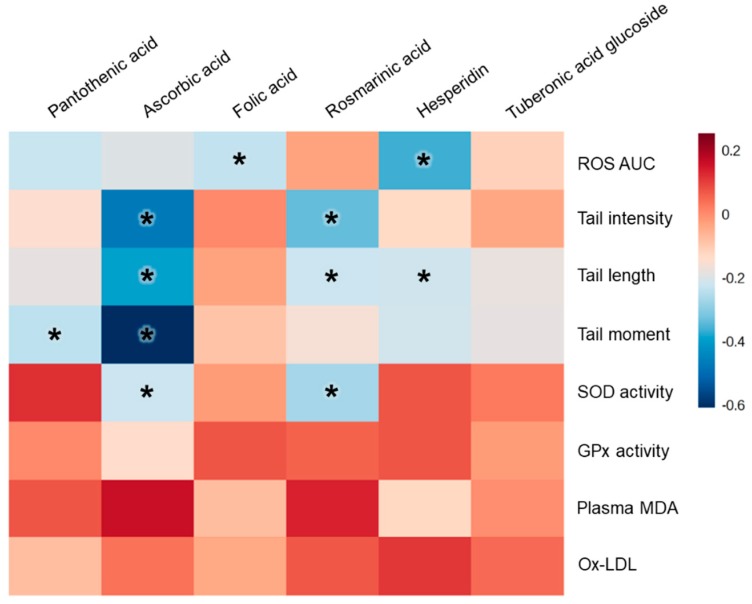
Heat map illustrating Pearson correlation coefficients between plasma and erythrocyte biomarkers and plasma vitamin and phytonutrients. Reactive oxygen species (ROS); area under the curve (AUC); superoxide dismutase (SOD); glutathione peroxidase (GPx); malondialdehyde (MDA); oxidized low-density lipoprotein (Ox-LDL). * *p* < 0.05.

One vitamin and 19 phytonutrients were identified in PMP product by UPLC-Q-TOF-MS and UHPLC-LTQ-IT-MS/MS analysis (Figure 5A and Table S6). Three vitamins and three phytonutrients were detected in the plasma by LC-triple-Q-MS analysis (Figure 5B). Pantothenic acid significantly increased (*p* = 0.007) in PMP compared to placebo group. All other phytonutrients and vitamins were not significantly different. The correlation between biomarkers and phytonutrients and vitamins analyzed in plasma are illustrated with a heat map (Figure 6) and r-values with *p*-values (Table 3). Plasma folic acid and hesperidin levels were negative correlated with ROS AUC (*p* < 0.05). Ascorbic acid and rosmarinic acid were negatively correlated with DNA tail intensity (*p* < 0.05). Plasma ascorbic acid, rosmarinic acid, and hesperidin levels were negatively correlated with tail length (*p* < 0.05). Plasma pantothenic acid and ascorbic acid levels were negatively correlated with tail moment (*p* < 0.05). Plasma rosmarinic acid was negatively correlated with SOD levels (*p* < 0.05).

## 4. Discussion

In our previous clinical trial, nutritional supplementation with a multivitamin and mineral containing phytonutrients was effective in reducing oxidative damage while maintaining endogenous ROS homeostasis [19]. In this trial, PMP supplementation reduced DNA damage without altering endogenous antioxidant enzyme activities, and increased ROS scavenging, which is consistent with our previous study. A certain amount of oxidative stress is useful to the body for growth and cell signaling, and our body has a defense system for controlling low level of oxidative stress such as glutathione, vitamin C, vitamin E, and antioxidant enzymes [29]. It has been reported that low grade level of oxidative stress is crucial to maintaining and priming our endogenous antioxidant system against high levels of oxidative stress and damage [30]. Based on our two human intervention studies, PMP supplementation was effective on scavenging ROS and preventing DNA damage without stimulating antioxidant enzymes.

Single-cell electrophoresis (also known as the comet assay) is widely used for measuring ROS-induced DNA damage and fragmentation [31]. There are several reports that DNA repair is enhanced by fruit and vegetables [32,33,34]. According to our previous study, comet assay on PBMC from subjects who had low RFS showed improved DNA repair after supplementation with antioxidant and phytonutrients [19]. In this current study, we showed that PMP supplementation reduced DNA tails length in PBMC. Although comet assay has been widely used for testing DNA repair, there is no standard reference value. Therefore, in this study, we tested comet assay before and after PMP and placebo supplementation and compared the changed values between placebo and PMP groups. In addition, compared to our previous study, we examined the protein expression of plasma pCHK1-Ser345 levels and detected modest increases in protein levels after PMP supplementation. Cellular responses to DNA repair are initiated by the ATR-CHK1 pathway, which is activated by single-stranded DNA breaks according to oxidative stress in a baseline of physiology status [35]. These data suggest that the PMP supplementation might provide protection against ROS-induced DNA damage by initiating single-strand break repair signaling responses via ATR-CHK1 pathway [36,37]. The effect of PMP supplements such as components of folic acid and ascorbic acid may be attributed in part to the induced pCHK1 protein expression of DNA damage and repair pathway [38].

To examine whether circulating oxidative stress genes were modulated by PMP supplementation, a qPCR array containing oxidative and inflammatory stress genes was performed on PBMC. PMP supplementation did not significantly stimulate or suppress genes involved in oxidative defenses. In addition, PMP supplementation did not alter the expressions of endogenous antioxidant genes. However, according to enriched network analysis, subtle changes of each gene might have induced relationships between genes and proteins with similar functions. In the PMP group compared to placebo, genes such as SOD2, CYBA, and CYBB were modestly upregulated and these genes are involved in superoxide metabolic processes. It has been reported that CYBB deficiency enhances multiple inflammatory cascades and deficiency of NCF1 ameliorates the disease [39].

Phytonutrients are known to be poorly absorbed in small intestine and many are metabolized in the gut mucosa and/or liver followed by conjugation to glucuronide, sulfate and/or methyl groups [40]. In addition, phytonutrients reaching the colon are extensively transformed by the microbiota and then excreted in bile and urine, usually within 24–48 h [16]. In this study, the levels of plasma phytonutrient were low and there is limited information on their metabolized forms. Rosmarinic acid and hesperidin have been reported to be metabolized by colonic bacteria [41,42]. When orally ingested, hesperidin cannot be metabolized by β-glucosidase in the small intestine but hydrolyzed to hesperetin aglycon by colonic microbiota [42]. Rosmarinic acid is known to be degraded into caffeic acid and 3-(3,4-dihydroxyphenyl)lactic acid [43,44]. Therefore, it was difficult to quantify phytonutrients in plasma as intact form. However, with limitation for obtaining standard compounds for each metabolite, in this study, several identified phytonutrients containing in PMP were analyzed in pooled plasma samples. Among phytonutrients, rosmarinic acid, hesperidin, and tuberonic acid glucoside, and among vitamins, pantothenic acid, ascorbic acid, and folic acid were negatively correlated with ROS and DNA damage. Rosmarinic acid and hesperidin showed significant negative correlation to tail intensity and length. In addition, hesperidin also showed significant negative correlation to ROS scavenging. Rosmarinic acid and hesperidin have been reported to prevent DNA damage and scavenge ROS; however, these studies are in vitro and animal studies [45,46,47,48,49,50]. Although mechanisms of action of these flavonoids and their metabolites could not be revealed in the present trial, this is the first clinical study reporting a relationship between the level of rosmarinic acid and hesperidin to DNA damage and ROS scavenging.

## 5. Conclusions

PMP supplementation for eight weeks reduced ROS and prevented DNA damage without altering endogenous antioxidant system, and several plasma vitamins and phytonutrients were significantly correlated with ROS scavenging and prevention of DNA damage. Gene expression analysis in PBMC after PMP supplementation showed subtle changes in superoxide metabolic processes.

## Figures and Tables

**Table 1:** Baseline characteristics of subjects ^1^.

Variable	Placebo	PMP	*p*-Value ^2^
	(*n* = 42)	(*n* = 42)	
Age (year)	41.6 ± 1.7	38.2 ± 1.7	0.169
Gender (male/female, *n*)	13/29	13/29	1.000
Recommended food score	19.5 ± 1.3	19.1 ± 1.5	0.830
Body weight (kg)	67.4 ± 2.1	65.1 ± 2.2	0.462
Body mass index (kg/m^2^)	24.8 ± 0.6	23.7 ± 0.6	0.202
Percent of body fat (%)	31.7 ± 0.9	30.1 ± 1.0	0.258
Smoker, *n* (%)	3 (7.1)	4 (9.5)	0.693
Alcohol drinker, *n* (%)	22 (52.4)	24 (57.1)	0.661
Blood pressure (mmHg)			
Systolic blood pressure	119.1 ± 2.1	116.7 ± 2.0	0.414
Diastolic blood pressure	79.5 ± 1.6	79.0 ± 1.5	0.786
Blood lipid profiles (mg/dL)			
Total triglyceride	142.2 ± 18.1	121.4 ± 9.2	0.311
Total cholesterol	189.5 ± 5.7	187.0 ± 4.3	0.729
LDL cholesterol	119.4 ± 5.7	120.6 ± 4.2	0.856
HDL cholesterol	53.2 ± 2.2	54.1 ± 1.8	0.752

^1^ Data are expressed as mean ± SE for continuous variables or as frequency and percentage for categorical variables. LDL, low density lipoprotein; HDL, high density lipoprotein. ^2^ Differences between the placebo and PMP groups were evaluated using the Student’s *t*-test for continuous variables and chi-square test for categorical variables.

**Table 2:** The effect of placebo and PMP supplementation on lipid oxidation and endogenous antioxidant defense ^1^.

Variable	Placebo (*n* = 42)		PMP (*n* = 42)		Estimate ^2^	*p*-Value
	Week 0	Week 8	Week 0	Week 8		
Erythrocyte						
SOD activity (U/mL)	200.25 ± 4.32	192.76 ± 3.63	205.87 ± 4.32	206.02 ± 3.63	7.637	0.250
GPx activity (µmol/min/mL)	1.12 ± 0.04	1.10 ± 0.04	1.11 ± 0.04	1.11 ± 0.04	0.017	0.559
Plasma						
MDA (µmol/L)	2.97 ± 0.14	2.99 ± 0.14	2.96 ± 0.14	3.02 ± 0.14	0.040	0.774
Oxidized LDL (U/L)	41.18 ± 1.84	39.89 ± 1.73	43.25 ± 1.84	41.78 ± 1.73	−0.176	0.914

^1^ All values are LS mean ± SE. superoxide dismutase (SOD); glutathione peroxidase (GPx); malondialdehyde (MDA). ^2^ Estimates were determined for each variable by calculating β, the estimated slope, from linear mixed-effects model. *p*-values were derived from a linear mixed-effects model. *p*-value < 0.05 was considered significant.

**Table 3:** Correlation between biomarkers and plasma vitamins and phytonutrients ^1^.

	Pantothenic Acid		Ascorbic Acid		Folic Acid		Rosmarinic Acid		Hesperidin		Tuberonic Acid Glucoside	
	r	*p* ^2^	r	*p*	r	*p*	r	*p*	r	*p*	r	*p*
ROS AUC	−0.227	0.0549	−0.194	0.1022	−0.233	0.0485	−0.029	0.8070	−0.365	0.0016	−0.113	0.3431
Tail intensity	−0.148	0.1969	−0.471	<0.0001	0.006	0.9618	−0.338	0.0025	−0.127	0.2680	−0.038	0.7397
Tail length	−0.187	0.0921	−0.390	0.0003	−0.032	0.7777	−0.221	0.0464	−0.217	0.0497	−0.178	0.1100
Tail moment	−0.238	0.0367	−0.608	<0.0001	−0.087	0.4529	−0.158	0.1713	−0.211	0.0659	−0.186	0.1061
SOD activity	0.121	0.2730	−0.222	0.0420	−0.019	0.8667	−0.268	0.0139	0.073	0.5088	0.026	0.8170
GPx activity	0.008	0.9417	−0.138	0.2109	0.074	0.5013	0.058	0.6020	0.073	0.5081	−0.021	0.8484
Plasma MDA	0.075	0.4964	0.165	0.1329	−0.073	0.5102	0.138	0.2118	−0.125	0.2578	−0.001	0.9919
Ox-LDL	−0.078	0.4786	0.036	0.7422	−0.039	0.7261	0.069	0.5358	0.111	0.3150	0.046	0.6794

^1^ Reactive oxygen species (ROS); area under the curve (AUC); superoxide dismutase (SOD); glutathione peroxidase (GPx); malondialdehyde (MDA); oxidized low-density lipoprotein (Ox-LDL). ^2^ *p*-values were calculated using Pearson correlation coefficient analysis.

## Supplementary Materials

The following are available online at http://www.mdpi.com/2072-6643/11/1/101/s1, Figure S1: Study design, Table S1: Inclusion and exclusion criteria, Table S2: Genes in PCR array, Table S3: Subjects dietary intake and lifestyles, Table S4: Change in gene expression in PBMC after eight-week supplementation, Table S5: List of top ten pathways changed after eight-week PMP supplementation (gene ontology analysis), Table S6: UPLC-Q-TOF-MS and UHPLC-LTQ-IT-MS/MS identified vitamin and phytonutrients in PMP study product.
