# Supplementary material for: Multivitamin and Mineral Supplementation Containing Phytonutrients Scavenges Reactive Oxygen Species in Healthy Subjects: A Randomized, Double-Blinded, Placebo-Controlled Trial
Source: Nutrients. 2019 Jan 5;11(1):101. doi: 10.3390/nu11010101 (PMC6356358; doi:10.3390/nu11010101)
Supplement: Supplementary file 1 [file nutrients-11-00101-s001.zip › Supplementary Figure.pptx]

## Slide 1
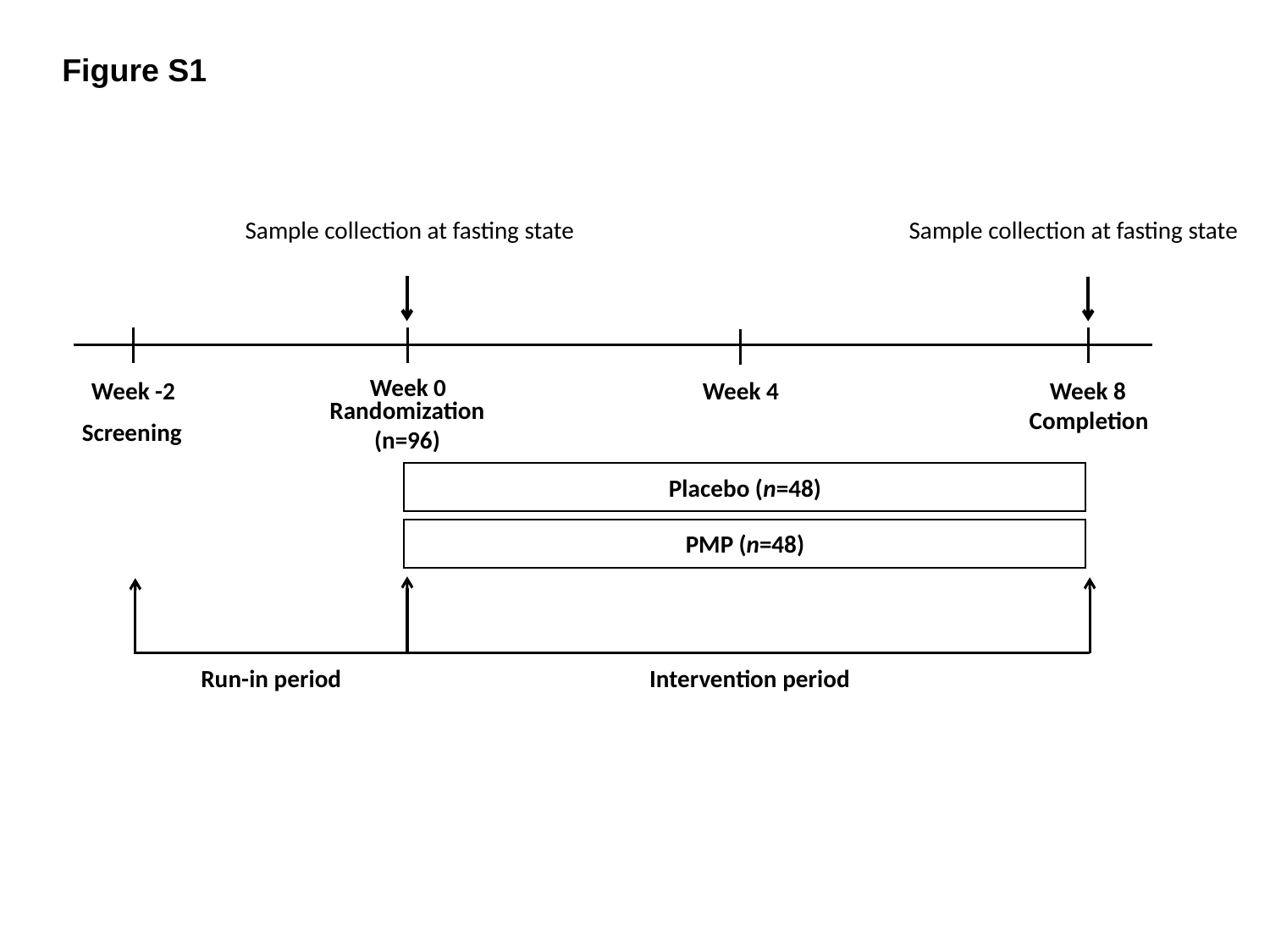

Figure S1
Sample collection at fasting state
Week 0
Week -2
Week 4
Week 8
Randomization
(n=96)
Screening
Completion
Placebo (n=48)
PMP (n=48)
Run-in period
Intervention period
Sample collection at fasting state
