# Supplementary material for: Multivitamin and Mineral Supplementation Containing Phytonutrients Scavenges Reactive Oxygen Species in Healthy Subjects: A Randomized, Double-Blinded, Placebo-Controlled Trial
Source: Nutrients. 2019 Jan 5;11(1):101. doi: 10.3390/nu11010101 (PMC6356358; doi:10.3390/nu11010101)
Supplement: Supplementary file 1 [file nutrients-11-00101-s001.zip › Supplementary table.docx]

**Supplementary Data**

**Table S1.** Inclusion and exclusion criteria.

| **Inclusion criteria** |
| --- |
| - - Men and women aged 25 to 69 years, with no more than 50% of all subjects younger than 40 years of age.   - Recommended Foods Score of 36 or less.   - Smokers and non-smokers will both be allowed into the study. The ratio of smokers to non-smokers will be matched between the placebo and the PMP groups. |
| **Exclusion criteria** |
| - - Use of supplements, vitamins, or herb remedies within eight weeks of the Screening visit.   - Body fat < 20%.   - Presence of ongoing medical illness or disease within two years before screening.     - Hypertension, diabetes, atherosclerosis, chronic inflammatory disease (rheumatoid arthritis, etc.), heart disease, kidney disease, liver disease, thyroid disease, malignant tumor, psychiatric disorder, etc.     - Subjects who had other illnesses that the principal investigator judged as meeting exclusion criteria.   - Taking medications within four weeks before screening: lipid-lowering agents, anti-inflammatory agents, antihypertensive drugs, blood glucose-lowering drugs, and antibiotics affecting the inflammatory response, antioxidant capacity, and lipid metabolism.   - Therapeutic uses of coumadin, aspirin, or other medications that influence hemostasis within four weeks of the Screening visit. Preventive use of low-dose aspirin (e.g. 100 mg/d) was allowed.   - A change in hormone therapy, including oral contraceptives, within four weeks prior to screening, or unwilling to maintain current hormone therapy/oral contraceptive use throughout the study period. |

**Table S1.** (continued).

| **Exclusion criteria** |
| --- |
| - - Weight loss of more than four kg within four weeks prior to screening visit or participating in a weight management program.   - History or current drug abuse or alcohol intake ≥ 420 g pure alcohol/week. (e.g. more than 7 bottles of soju/week).   - Unwilling to maintain present exercise and dietary habits and lifestyles for the duration of the study. (e.g. excessive exercise ≥ seven h/week, excessive smoking ≥ ten cigarettes/day)   - Known hypersensitivity to any ingredients in study products.   - Participation in another clinical trial within four weeks of enrollment into the study.   - Blood donation within past four weeks prior to screening.   - Pregnant or lactating women.   - Any condition that the Principal Investigator believes may put the subject at undue risk. |

**Table S2.** Genes in PCR array.

| **Biological function** | **Target gene** | **Full name** |
| --- | --- | --- |
| **Housekeeping gene** | GAPDH | Glyceraldehyde 3-phosphate dehydrogenase |
| **Inflammatory mediators** | AKT1 | Akt murine thymoma viral oncogene homolog 1 |
| **and signaling molecules** | BCL2 | B-cell CLL/lymphoma 2 |
|  | BCL6 | B-cell CLL/lymphoma 6 |
|  | CCL2 | (MCP1) Chemokine (C-C motif) ligand 2 |
|  | CCL22 | Chemokine (C-C motif) ligand 22 |
|  | CCL3 | (MIP-1a) Chemokine (C-C motif) ligand 3 |
|  | CCL4 | Chemokine (C-C motif) ligand 4 |
|  | CCL5 | (RANTES) Chemokine (C-C motif) ligand 5 |
|  | CCR3 | Chemokine (C-C motif) receptor 3 |
|  | CD40 | CD40 molecule |
|  | CD80 | CD80 molecule |
|  | CD86 | CD86 molecule |
|  | CR1 | Complement component (3b/4b) receptor 1 (Knops blood group) |
|  | CRLF1 | Cytokine receptor-like factor 1 |
|  | CXCL10 | Chemokine (C-X-C motif) ligand 10 |
|  | CXCL11 | Chemokine (C-X-C motif) ligand 11 |
|  | CXCL8 | Chemokine (C-X-C motif) ligand 8 |
|  | CXCL9 | Chemokine (C-X-C motif) ligand 9 |
|  | EGFR | Epidermal growth factor receptor |
|  | ESR1 | Estrogen receptor 1 |
|  | GRB2 | Growth factor receptor-bound protein 2 |
|  | IFNA1 | Interferon, alpha 1 |
|  | IFNAR1 | Interferon (alpha, beta and omega) receptor 1 |
|  | IFNB1 | Interferon, beta 1, fibroblast |
|  | IL12A | Interleukin 12A |
|  | IL12B | Interleukin 12B |
|  | IL1B | Interleukin 1, beta |
|  | IL1R1 | Interleukin 1 receptor, type I |
|  | IL6 | Interleukin 6 (interferon, beta 2) |
|  | IRF3 | Interferon regulatory factor 3 |
|  | IRF7 | Interferon regulatory factor 7 |
|  | ITGAL | Integrin, alpha L (antigen CD11A (p180), lymphocyte function-associated antigen 1; alpha polypeptide) |
|  | LITAF | Lipopolysaccharide-induced TNF factor |
|  | MIF | Macrophage migration inhibitory factor (glycosylationinhibiting factor) |
|  | NFKBIA | Nuclear factor of kappa light polypeptide gene enhancer in B-cells inhibitor, alpha |
|  | PTGDR | Prostaglandin D2 receptor |
|  | PTPN6 | Protein tyrosine phosphatase, non-receptor type 6 |
|  | SELL | Selectin L |
|  | SIRPA | Signal-regulatory protein alpha |

**Table S2.** (continued).

| **Biological function** | **Target gene** | **Full name** |
| --- | --- | --- |
| **Inflammatory mediators** | SOCS3 | Suppressor of cytokine signaling 3 |
| **and signaling molecules** | SOCS6 | Suppressor of cytokine signaling 6 |
|  | STAT3 | Signal transducer and activator of transcription 3 (acutephase response factor) |
|  | TLR2 | Toll-like receptor 2 |
|  | TLR4 | Toll-like receptor 4 |
|  | TNF | Tumor necrosis factor |
|  | TNFRSF18 | Tumor necrosis factor receptor superfamily, member 18 |
|  | TNFRSF1B | Tumor necrosis factor receptor superfamily, member 1B |
| **Plaque formation** | ICAM1 | Intercellular adhesion molecule 1 |
| **and coagulation** | PEAR1 | Platelet endothelial aggregation receptor 1 |
|  | VCAM1 | Vascular cell adhesion molecule 1 |
| **Antioxidant** | AGER | Advanced glycosylation end product-specific receptor |
|  | CAT | Catalase |
|  | CYBA | Cytochrome b-245, alpha polypeptide |
|  | CYBB | Cytochrome b-245, beta polypeptide |
|  | GPX1 | Glutathione peroxidase 1 |
|  | GPX3 | Glutathione peroxidase 3 (plasma) |
|  | GPX4 | Glutathione peroxidase 4 |
|  | HMOX1 | Heme oxygenase (decycling) 1 |
|  | NCF1 | Neutrophil cytosolic factor 1 |
|  | NFE2L2 | Nuclear factor, erythroid 2-like 2 |
|  | NOS2 | Nitric oxide synthase 2, inducible |
|  | SOD1 | Superoxide dismutase 1, soluble |
|  | SOD2 | Superoxide dismutase 2, mitochondrial |
|  | TXNRD1 | Thioredoxin reductase 1 |
| **Blood cell differentiation** | CCL21 | Chemokine (C-C motif) ligand 21 |
|  | IL4R | Interleukin 4 receptor |
| **Lipid/lipoprotein** | ACACB | Acetyl-CoA carboxylase beta |
| **metabolism** | ACADSB | Acyl-CoA dehydrogenase, short/branched chain |
|  | APOE | Apolipoprotein E |
|  | DGAT1 | Diacylglycerol O-acyltransferase 1 |
|  | DGKA | Diacylglycerol kinase, alpha 80kDa |
|  | DGKH | Diacylglycerol kinase, eta |
|  | DGKQ | Diacylglycerol kinase, theta 110kDa |
|  | ECI1 | Enoyl-CoA delta isomerase 1 |
|  | GK | Glycerol kinase |
|  | GPAT4 | Glycerol-3-Phosphate Acyltransferase 4 |
|  | HADHA | Hydroxyacyl-CoA dehydrogenase/3-ketoacyl-CoA thiolase/enoyl-CoA hydratase (trifunctional protein), alpha subunit |
|  | HMGCR | 3-hydroxy-3-methylglutaryl-CoA reductase |
|  | LDLR | Low density lipoprotein receptor |
|  | LPAR2 | Lysophosphatidic acid receptor 2 |
|  | LRP1 | Low density lipoprotein receptor-related protein 1 |

**Table S2.** (continued).

| **Biological function** | **Target gene** | **Full name** |
| --- | --- | --- |
| **Lipid/lipoprotein  metabolism** | MOGAT3 | Monoacylglycerol O-acyltransferase 3 |
|  | NOX5 | NADPH oxidase 5 |
|  | PLB1 | Phospholipase B1 |
|  | PTDSS1 | Phosphatidylserine synthase 1 |
|  | RSAD2 | Radical S-adenosyl methionine domain containing 2 |
|  | SCD | Stearoyl-CoA desaturase (delta-9-desaturase) |
|  | PLB1 | Phospholipase B1 |
|  | PTDSS1 | Phosphatidylserine synthase 1 |
|  | RSAD2 | Radical S-adenosyl methionine domain containing 2 |
|  | SCD | Stearoyl-CoA desaturase (delta-9-desaturase) |
|  | SLC27A1 | Solute carrier family 27 (fatty acid transporter), member 1 |

**Table S3.** Subjects dietary intake and lifestyles ^1^.

| **Variable** | **Placebo (*n*=42)** | | | **PMP (*n*=42)** | | | ***P*-value^2^** |
| --- | --- | --- | --- | --- | --- | --- | --- |
| Energy (kcal/d) | 1778.3 | ± | 42.6 | 1782.5 | ± | 56.0 | 0.953 |
| Carbohydrate (g/d) | 264.1 | ± | 6.7 | 255.6 | ± | 7.9 | 0.417 |
| Protein (g/d) | 69 | ± | 2.2 | 71.5 | ± | 2.4 | 0.438 |
| Lipid (g/d) | 48.5 | ± | 2.0 | 52 | ± | 2.4 | 0.264 |
| **Vitamins** |  |  |  |  |  |  |  |
| Vitamin A (μg RE/d) | 631.8 | ± | 44.8 | 611.7 | ± | 31.1 | 0.713 |
| Retinol (μg/d) | 135.3 | ± | 10.6 | 140.9 | ± | 10.5 | 0.710 |
| β-carotene (μg/d) | 3204.8 | ± | 247.2 | 3072.5 | ± | 179.7 | 0.666 |
| Vitamin D (μg/d) | 3.8 | ± | 0.4 | 3.2 | ± | 0.3 | 0.240 |
| Vitamin E (mg/d) | 13.7 | ± | 0.7 | 15.2 | ± | 1.1 | 0.247 |
| Vitamin K (μg/d) | 138.8 | ± | 9.3 | 131.2 | ± | 8.5 | 0.548 |
| Vitamin C (mg/d) | 68.2 | ± | 4.3 | 58.7 | ± | 3.5 | 0.091 |
| Vitamin B1 (mg/d) | 1.3 | ± | 0.0 | 1.2 | ± | 0.0 | 0.824 |
| Vitamin B2 (mg/d) | 1.1 | ± | 0.0 | 1.2 | ± | 0.0 | 0.469 |
| Niacin (mg/d) | 15.7 | ± | 0.5 | 15.2 | ± | 0.6 | 0.497 |
| Vitamin B6 (mg/d) | 1.4 | ± | 0.1 | 1.3 | ± | 0.1 | 0.521 |
| Folate (μg/d) | 366.6 | ± | 18.3 | 344 | ± | 14.9 | 0.342 |
| Vitamin B12 (μg/d) | 8.4 | ± | 0.5 | 8.5 | ± | 0.6 | 0.982 |
| Pantothenic Acid (mg/d) | 4.1 | ± | 0.1 | 4 | ± | 0.1 | 0.751 |
| Biotin (μg/d) | 15.3 | ± | 0.7 | 16 | ± | 0.8 | 0.489 |
| **Minerals** |  |  |  |  |  |  |  |
| Calcium (mg/d) | 397.7 | ± | 17.6 | 379 | ± | 18.7 | 0.469 |
| Phosphorus (mg/d) | 953.8 | ± | 30.9 | 943.4 | ± | 34.4 | 0.823 |
| Sodium (mg/d) | 3942.7 | ± | 167.2 | 4170.4 | ± | 167.4 | 0.339 |
| Chlorine (mg/d) | 379.3 | ± | 37.2 | 408.6 | ± | 39.6 | 0.590 |
| Potassium (mg/d) | 2221.5 | ± | 82.2 | 2160.6 | ± | 79.7 | 0.596 |
| Magnesium (mg/d) | 73.5 | ± | 3.3 | 75 | ± | 3.6 | 0.767 |
| Iron (mg/d) | 16.5 | ± | 0.8 | 16.6 | ± | 1.0 | 0.920 |
| Zinc (mg/d) | 9 | ± | 0.3 | 9.2 | ± | 0.4 | 0.634 |
| Copper (mg/d) | 1 | ± | 0.0 | 1 | ± | 0.0 | 0.351 |
| Fluorine (μg/d) | 26.8 | ± | 4.9 | 17.6 | ± | 1.9 | 0.088 |
| Manganese (mg/d) | 3.1 | ± | 0.1 | 2.9 | ± | 0.1 | 0.330 |
| Iodine (μg/d) | 285.1 | ± | 43.1 | 241 | ± | 40.8 | 0.460 |
| Selenium (μg/d) | 99.6 | ± | 3.8 | 106.9 | ± | 6.0 | 0.310 |
| **Carotenoids (μg/d)** |  |  |  |  |  |  |  |
| α-carotene | 669.2 | ± | 63.0 | 785 | ± | 54.8 | 0.169 |
| β-carotene | 5019.7 | ± | 569.1 | 4223.1 | ± | 386.9 | 0.251 |
| β-cryptoxanthin | 245.2 | ± | 33.8 | 144.3 | ± | 24.4 | 0.018 |
| Lutein + zeaxanthin | 2056.4 | ± | 154.0 | 2067.4 | ± | 152.3 | 0.960 |
| Lycopene | 465.9 | ± | 60.2 | 440.3 | ± | 74.2 | 0.789 |
| Total carotenoid | 8456.3 | ± | 682.0 | 7660.1 | ± | 497.1 | 0.349 |

**Table S3.** (continued).

| **Variable** | **Placebo (*n*=42)** | | | **PMP (*n*=42)** | | | ***P*-value^2^** |
| --- | --- | --- | --- | --- | --- | --- | --- |
| **Flavonoids (mg/d)** |  |  |  |  |  |  |  |
| Anthocyanidins | 9.8 | ± | 1.2 | 8.5 | ± | 1.1 | 0.422 |
| Flavan-3-ols | 7.3 | ± | 1.7 | 6.8 | ± | 1.6 | 0.857 |
| Flavanones | 4.7 | ± | 0.8 | 2.4 | ± | 0.6 | 0.026 |
| Flavones | 0.6 | ± | 0.1 | 0.5 | ± | 0.1 | 0.689 |
| Flavonols | 15 | ± | 1.3 | 15.7 | ± | 0.9 | 0.671 |
| Isoflavones | 15.9 | ± | 1.5 | 15.4 | ± | 1.3 | 0.806 |
| Total flavonoid | 53.2 | ± | 3.5 | 49.4 | ± | 3.5 | 0.431 |
| **Lifestyles** |  |  |  |  |  |  |  |
| Alcohol amount (g/week) | 20.0 | ± | 4.9 | 25.9 | ± | 6.4 | 0.467 |
| Physical activity level  (MET-min/week) | 1702.3 | ± | 173.7 | 1438.0 | ± | 208.9 | 0.334 |
| Total sleep time  (hours/week) | 48.9 | ± | 0.9 | 50.7 | ± | 0.9 | 0.169 |

^1^ All values are mean ± standard error (SE). MET, metabolic equivalent of task.

^2^ Significant difference between the placebo and PMP group was evaluated using student’s t-test.

**Table S4.** Change in gene expression in PBMC after eight-week supplementation^1^

| **Biological function** | **Target gene** | **Placebo** | | | **PMP** | | | ***p*-value^2^** |
| --- | --- | --- | --- | --- | --- | --- | --- | --- |
| **Inflammatory mediators** | AKT1 | 0.8 | ± | 0.4 | 1.9 | ± | 0.4 | 0.589 |
| **and signaling molecules** | BCL2 | 0.9 | ± | 0.2 | 1.4 | ± | 0.2 | 0.436 |
|  | BCL6 | 0.9 | ± | 0.2 | 1.5 | ± | 0.2 | 0.428 |
|  | CCL2 | 0.8 | ± | 0.4 | 1.9 | ± | 0.4 | 0.143 |
|  | CCL22 | 0.5 | ± | 0.2 | 0.9 | ± | 0.2 | 0.829 |
|  | CCL3 | 0.5 | ± | 0.2 | 1.4 | ± | 0.2 | 0.133 |
|  | CCL4 | 0.8 | ± | 0.2 | 1.2 | ± | 0.2 | 0.641 |
|  | CCL5 | 1.0 | ± | 0.2 | 1.1 | ± | 0.2 | 0.675 |
|  | CCR3 | 1.9 | ± | 0.4 | 1.3 | ± | 0.4 | 0.395 |
|  | CD40 | 1.0 | ± | 0.2 | 1.4 | ± | 0.2 | 0.443 |
|  | CD80 | 1.4 | ± | 0.3 | 1.9 | ± | 0.3 | 0.904 |
|  | CD86 | 0.6 | ± | 0.1 | 0.7 | ± | 0.1 | 0.988 |
|  | CR1 | 1.6 | ± | 0.3 | 1.7 | ± | 0.3 | 0.920 |
|  | CRLF1 | 0.9 | ± | 0.4 | 1.5 | ± | 0.4 | 0.656 |
|  | CXCL10 | 0.9 | ± | 0.4 | 1.3 | ± | 0.4 | 0.500 |
|  | CXCL11 | 1.3 | ± | 0.3 | 0.8 | ± | 0.3 | 0.318 |
|  | CXCL8 | 0.8 | ± | 0.3 | 0.8 | ± | 0.3 | 0.747 |
|  | CXCL9 | 1.0 | ± | 0.4 | 1.7 | ± | 0.4 | 0.520 |
|  | EGFR | 1.7 | ± | 0.5 | 1.5 | ± | 0.5 | 0.895 |
|  | ESR1 | 1.5 | ± | 0.2 | 0.3 | ± | 0.2 | 0.118 |
|  | GRB2 | 1.3 | ± | 0.2 | 1.6 | ± | 0.2 | 0.449 |
|  | IFNA1 | 0.9 | ± | 0.4 | 1.6 | ± | 0.3 | 0.432 |
|  | IFNAR1 | 1.2 | ± | 0.5 | 1.9 | ± | 0.5 | 0.517 |
|  | IFNB1 | 1.6 | ± | 0.4 | 1.0 | ± | 0.4 | 0.524 |
|  | IL12A | 0.9 | ± | 0.4 | 2.0 | ± | 0.4 | 0.151 |
|  | IL12B | 0.9 | ± | 0.5 | 2.4 | ± | 0.5 | 0.202 |
|  | IL1B | 1.3 | ± | 0.4 | 1.9 | ± | 0.4 | 0.386 |
|  | IL1R1 | 1.3 | ± | 0.3 | 1.0 | ± | 0.4 | 0.673 |
|  | IL6 | 0.7 | ± | 0.3 | 1.5 | ± | 0.3 | 0.393 |
|  | IRF3 | 0.9 | ± | 0.3 | 1.4 | ± | 0.2 | 0.191 |
|  | IRF7 | 1.3 | ± | 0.4 | 1.8 | ± | 0.4 | 0.782 |
|  | ITGAL | 0.9 | ± | 0.2 | 1.3 | ± | 0.2 | 0.666 |
|  | LITAF | 0.7 | ± | 0.2 | 0.7 | ± | 0.2 | 0.972 |
|  | MIF | 1.2 | ± | 0.2 | 0.8 | ± | 0.2 | 0.112 |
|  | NFKBIA | 1.3 | ± | 0.2 | 1.3 | ± | 0.2 | 0.992 |
|  | PTGDR | 1.2 | ± | 0.2 | 1.3 | ± | 0.2 | 0.972 |
|  | PTPN6 | 0.8 | ± | 0.1 | 1.3 | ± | 0.1 | 0.394 |
|  | SELL | 1.8 | ± | 0.5 | 1.0 | ± | 0.5 | 0.171 |
|  | SIRPA | 1.0 | ± | 0.3 | 1.8 | ± | 0.3 | 0.262 |
|  | SOCS3 | 0.8 | ± | 0.3 | 1.3 | ± | 0.2 | 0.557 |
|  | SOCS6 | 1.6 | ± | 0.7 | 2.3 | ± | 0.7 | 0.512 |
|  | STAT3 | 0.9 | ± | 0.2 | 1.2 | ± | 0.2 | 0.928 |

**Table S4.** (continued).

| **Biological function** | **Target gene** | **Placebo** | | | **PMP** | | | ***p*-value^2^** |
| --- | --- | --- | --- | --- | --- | --- | --- | --- |
| **Inflammatory mediators** | TLR2 | 1.0 | ± | 0.2 | 1.7 | ± | 0.2 | 0.054 |
| **and signaling molecules** | TLR4 | 1.4 | ± | 0.4 | 1.5 | ± | 0.4 | 0.927 |
|  | TNF | 1.3 | ± | 0.3 | 1.9 | ± | 0.3 | 0.735 |
|  | TNFRSF18 | 1.4 | ± | 0.3 | 1.6 | ± | 0.3 | 0.206 |
|  | TNFRSF1B | 0.9 | ± | 0.4 | 1.3 | ± | 0.4 | 0.314 |
| **Plaque formation** | ICAM1 | 0.8 | ± | 0.4 | 2.4 | ± | 0.4 | 0.069 |
| **and coagulation** | PEAR1 | 1.1 | ± | 0.5 | 2.0 | ± | 0.4 | 0.390 |
|  | VCAM1 | 0.7 | ± | 0.3 | 1.1 | ± | 0.3 | 0.710 |
| **Antioxidant** | AGER | 0.9 | ± | 0.3 | 1.3 | ± | 0.3 | 0.580 |
|  | CAT | 0.4 | ± | 0.2 | 1.1 | ± | 0.2 | 0.335 |
|  | CYBA | 0.9 | ± | 0.3 | 1.3 | ± | 0.3 | 0.769 |
|  | CYBB | 0.9 | ± | 0.2 | 1.2 | ± | 0.2 | 0.454 |
|  | GPX1 | 1.0 | ± | 0.2 | 0.7 | ± | 0.2 | 0.516 |
|  | GPX3 | 1.3 | ± | 0.5 | 1.8 | ± | 0.4 | 0.769 |
|  | GPX4 | 0.8 | ± | 0.1 | 1.1 | ± | 0.1 | 0.547 |
|  | HMOX1 | 0.7 | ± | 0.1 | 1.2 | ± | 0.1 | 0.635 |
|  | NCF1 | 0.8 | ± | 0.3 | 1.2 | ± | 0.3 | 0.548 |
|  | NFE2L2 | 0.7 | ± | 0.1 | 1.0 | ± | 0.1 | 0.851 |
|  | SOD1 | 1.3 | ± | 0.2 | 1.7 | ± | 0.2 | 0.836 |
|  | SOD2 | 1.1 | ± | 0.2 | 1.4 | ± | 0.2 | 0.333 |
|  | TXNRD1 | 0.9 | ± | 0.2 | 0.7 | ± | 0.2 | 0.579 |
| **Blood cell differentiation** | CCL21 | 0.8 | ± | 0.4 | 1.7 | ± | 0.3 | 0.486 |
|  | IL4R | 0.7 | ± | 0.2 | 1.2 | ± | 0.2 | 0.390 |
| **Lipid/lipoprotein** | ACACB | 1.1 | ± | 0.1 | 1.1 | ± | 0.1 | 0.883 |
| **metabolism** | ACADSB | 1.5 | ± | 0.3 | 1.4 | ± | 0.3 | 0.940 |
|  | APOE | 1.3 | ± | 0.4 | 1.8 | ± | 0.4 | 0.490 |
|  | DGAT1 | 1.1 | ± | 0.3 | 1.1 | ± | 0.3 | 0.966 |
|  | DGKA | 1.1 | ± | 0.3 | 0.9 | ± | 0.3 | 0.875 |
|  | DGKH | 0.4 | ± | 0.3 | 2.1 | ± | 0.3 | 0.070 |
|  | DGKQ | 0.5 | ± | 0.3 | 1.7 | ± | 0.3 | 0.368 |
|  | ECI1 | 0.8 | ± | 0.3 | 1.5 | ± | 0.3 | 0.313 |
|  | GK | 1.3 | ± | 0.3 | 1.5 | ± | 0.3 | 0.619 |
|  | GPAT4 | 0.9 | ± | 0.2 | 1.7 | ± | 0.2 | 0.111 |
|  | HADHA | 0.8 | ± | 0.2 | 0.9 | ± | 0.2 | 0.951 |
|  | HMGCR | 1.5 | ± | 0.3 | 1.6 | ± | 0.3 | 0.967 |
|  | LDLR | 1.2 | ± | 0.6 | 2.4 | ± | 0.5 | 0.199 |
|  | LPAR2 | 1.4 | ± | 0.3 | 1.6 | ± | 0.3 | 0.692 |
|  | LRP1 | 1.0 | ± | 0.1 | 2.0 | ± | 0.1 | 0.113 |
|  | MOGAT3 | 0.8 | ± | 0.4 | 1.8 | ± | 0.4 | 0.325 |
|  | NOX5 | 0.5 | ± | 0.3 | 1.6 | ± | 0.3 | 0.417 |
|  | PLB1 | 0.5 | ± | 0.4 | 2.3 | ± | 0.4 | 0.119 |
|  | PTDSS1 | 1.6 | ± | 0.4 | 1.8 | ± | 0.4 | 0.559 |

**Table S4.** (continued).

| **Biological function** | **Target gene** | **Placebo** | | | **PMP** | | | ***p*-value^2^** |
| --- | --- | --- | --- | --- | --- | --- | --- | --- |
| **Lipid/lipoprotein** | RSAD2 | 1.3 | ± | 0.5 | 1.8 | ± | 0.5 | 0.492 |
| **metabolism** | SCD | 0.7 | ± | 0.5 | 2.1 | ± | 0.4 | 0.139 |
|  | SLC27A1 | 1.6 | ± | 0.5 | 2.1 | ± | 0.5 | 0.756 |

^1^ Changes are relative to baseline values. Data are presented as LS means ± SE.

^2^ *p*-values were derived from a linear mixed-effects model.

**Table S5.** List of top ten pathways changed after eight-week PMP supplementation (gene ontology analysis).

| **Annotation (pathway/process)** | **Fisher q-value** |
| --- | --- |
| GO:0042554~superoxide anion generation | 0.000453875 |
| GO:0042832~defense response to protozoan | 0.008365847 |
| GO:0019953~sexual reproduction | 0.008365847 |
| GO:0045730~respiratory burst | 0.008365847 |
| GO:0051881~regulation of mitochondrial membrane potential | 0.009776462 |
| GO:0006801~superoxide metabolic process | 0.000779746 |
| GO:0043922~negative regulation by host of viral transcription | 0.011237552 |
| GO:0010332~response to gamma radiation | 0.000003546 |
| GO:0042100~B cell proliferation | 0.012034604 |
| GO:0014911~positive regulation of smooth muscle cell migration | 0.012034604 |

**Table S6.** UPLC-Q-TOF-MS and UHPLC-LTQ-IT-MS/MS identified vitamin and phytonutrients in PMP study product.

| No. | UPLC-Q-TOF-MS | | | | | | UHPLC-LTQ-MS/MS |  |
| --- | --- | --- | --- | --- | --- | --- | --- | --- |
|  | RT | Tentative identification | Measured ion Adduct | | VIP | Formula | MS fragments | |
| 1 | 0.88 | Proanthocyanidin derivatives | 577.1355 | [M-H]^-^ | 1.17 | C_30_H_25_O_12_ | 577>559>533>467>425>409>391>205>289>245 | |
| 2 | 2.33 | Pantothenic acid | 220.1148 | [M+H]^+^ | 1.15 | C_9_H_18_NO_5_ | 220>202>184>174>116>90 | |
| 3 | 3.00 | Cyanidin 3-*O*-rutinoside | 595.1675 | [M+H]^+^ | 1.17 | C_27_H_31_O_15_ | 595>577>449>427>409>287 | |
| 4 | 3.41 | Tuberonic acid glucoside | 387.1651 | [M-H]^-^ | 1.17 | C_18_H_27_O_9_ | 387>369>341>207>163>145 | |
| 5 | 3.76 | Rutin | 611.1686 | [M+H]^+^ | 1.16 | C_27_H_31_O_16_ | 611>593>464>449>431>303 | |
| 6 | 3.80 | Kaemperol-rutinoside | 593.1524 | [M-H]^-^ | 1.17 | C_27_H_29_O_15_ | 593>575>282>257>229 | |
| 7 | 3.90 | Quercetin-3-*O*-glucoside | 463.0892 | [M-H]^-^ | 1.14 | C_21_H_19_O_12_ | 463>343>301>271>191 | |
| 8 | 3.96 | Sudachiin A | 521.1332 | [M-H]^-^ | 1.17 | C_24_H_25_O_13_ | 521>359>323>224 | |
| 9 | 3.99 | Quercetin-diglucoside | 625.1263 | [M-H]^-^ | 1.17 | C_27_H_30_O_17_ | 625>579>301 | |
| 10 | 4.18 | Naringin | 579.1719 | [M-H]^-^ | 1.10 | C_27_H_31_O_14_ | 579>459>417>313>271>235 | |
| 11 | 4.26 | Hesperidin | 609.1667 | [M-H]^-^ | 1.17 | C_28_H_33_O_15_ | 609>563>301>271 | |
| 12 | 4.38 | Rosmarinic acid | 359.0763 | [M-H]^-^ | 1.17 | C_18_H_15_O_8_ | 359>223>197>179>161 | |
| 13 | 4.60 | Petunidin glycoside glucuronides | 655.1708 | [M+H]^+^ | 1.17 | C_32_H_31_O_15_ | 655>479>339>317>302>287 | |
| 14 | 4.66 | Glucosyl–rhamnosyl isorhamnetin | 623.1417 | [M-H]^-^ | 1.17 | C_31_H_27_O_14_ | 623>608>477>315>300 | |
| 15 | 4.99 | Quercetin | 303.0359 | [M+H]^+^ | 1.15 | C_15_H_11_O_7_ | 303>285>274>257>229>165>153>137 | |
| 16 | 5.16 | Peonidin 3-glucoside | 463.1299 | [M+H]^+^ | 1.17 | C_22_H_23_O_11_ | 463>301>287 | |
| 17 | 5.41 | Phloretin | 273.0711 | [M-H]^-^ | 1.17 | C_15_H_13_O_5_ | 273>254>189>167 | |
| 18 | 5.57 | Demethoxycurcumin | 337.1071 | [M-H]^-^ | 1.17 | C_20_H_17_O_5_ | 337>217>187>173>143>119 | |
| 19 | 5.65 | Curcumin | 367.1174 | [M-H]^-^ | 1.17 | C_21_H_19_O_6_ | 367>217>173>149>135 | |
| 20 | 6.12 | Wogonin | 283.0242 | [M-H]^-^ | 1.17 | C_15_H_7_O_6_ | 283>268>255>240 | |
| 21 | 6.89 | Curcumin | 369.1187 | [M+H]^+^ | 1.17 | C_21_H_21_O_6_ | 369>299>285>245>175 | |
| 22 | 8.67 | Carnosic acid | 331.1906 | [M-H]^-^ | 1.08 | C_20_H_27_O_4_ | 331>315>287>244 | |

Retention Time (RT); Variable importance in projection (VIP).
